# Isolated skeletal Langerhans cell histiocytosis in adults: A case report

**DOI:** 10.1016/j.ijscr.2024.109801

**Published:** 2024-05-25

**Authors:** Yuni Artha Prabowo Putro, Rahadyan Magetsari, Muhammad Ichwan Noorrafiqi, Ery Kus Dwianingsih, Ericko Ekaputra, Amri Wicaksono Pribadi

**Affiliations:** a Department of Orthopedics and Traumatology, RSUP Dr. Sardjito Hospital, Jl. Kesehatan Sendowo No.1, Sleman 55281, D.I.Yogyakarta, Indonesia; b Faculty of Medicine, Public Health and Nursing, Universitas Gadjah Mada, Jl. Farmako, Sendowo, Sekip Utara, Sleman 55281, D.I.Yogyakarta, Indonesia; c Department of Anatomical Pathology, Faculty of Medicine, Public Health and Nursing, Universitas Gadjah Mada, Dr Sardjito General Hospital, Jl. Farmako, Sendowo, Sekip Utara, Sleman 55281, D.I.Yogyakarta, Indonesia; d Department of Radiology, Faculty of Medicine, Public Health and Nursing, Universitas Gadjah Mada, Jl. Farmako, Sendowo, Sekip Utara, Sleman 55281, D.I.Yogyakarta, Indonesia

**Keywords:** Langerhans cell histiocytosis, Orthopedic oncology, Bone tumor, Skeletal lesion

## Abstract

**Introduction and importance:**

LCH in adults is rarely encountered, with the preference in children and axial skeleton as predilection site. Limited understanding of adult LCH causes frequent misdiagnosis, as our experience in an adult case of LCH threw off our differential diagnosis.

**Case presentation:**

A 21-year-old male was referred to our hospital due to pain in his right shoulder. Plain radiograph and MRI showed a solitary well-marginated lytic lesion on the distal third of the clavicle. Together with a clear history and physical exam, the benign bone cyst was suspected and we performed an open biopsy simultaneously with curettage followed by internal fixation using a bone graft. Pathology and immunohistochemistry dismissed our suspicion and confirmed LCH as the main diagnosis. At six months post-surgery, no signs of recurrence were seen on the fixated site nor complained by the patient.

**Discussion:**

Diagnosing LCH involves considering imaging appearances and patient demographics as initial clues. However, confirming the diagnosis requires a biopsy with proven CD1 expression. Currently, the majority of studies recommend confirming the diagnosis before initiating therapy. This precaution is necessary due to the unclear pathophysiology of LCH, which complicates the implementation of specific therapies**.** Based on benign features of skeletal lesions found from imaging, invasive treatment before biopsy confirmation still gave a satisfactory outcome despite not being in line with the current recommendation.

**Conclusion:**

Excisional biopsy and curettage in solitary LCH yield satisfactory outcomes. However, further studies are needed with larger sample sizes and interventional designs.

## Highlights

• LCH in adults is rarely encountered, with the preference in children and axial skeleton as predilection site.
• Limited understanding of adult LCH causes frequent misdiagnosis.
• Satisfactory outcomes were achieved by opting for invasive treatment before biopsy confirmation.

## 1 Introduction

Langerhans cell histiocytosis (LCH) refers to a spectrum of diseases characterized by the idiopathic proliferation of histiocytes, specifically Langerhans cells (LCs). The causes and pathogenesis of LCH remain unknown, although theories of immune dysregulation and genetic involvement have been proposed, albeit still under debate.

LCH primarily affects children up to the age of 15, making its occurrence in adults uncommon and poorly understood. In adults, LCH is reported in fewer than 0.1 cases per million, compared to 9 cases per million in children under 15 years old annually [[1], [2], [3]]. Consequently, LCH is not typically suspected as the cause of osteolytic lesions in adults, given its higher prevalence in children. However, if diagnosed in adults, it tends to manifest in specific areas such as the skull, mandible, and axial skeleton. There have been a few reported cases of LCH in adults with single lesions [[4], [5], [6], [7], [8]].

Clinical manifestations of LCH vary across the spectrum of diseases and further depend on the age of presentation. Skeletal involvement is most commonly reported in up to 80 % of LCH patients, with a higher incidence in pediatric populations than in adults, typically manifesting in the skull, spine, limbs, and pelvis [3,4]. Imaging often reveals osteolytic lesions due to dendritic cell infiltration. Pathologically, lesions from LCH diseases consist uniformly of bone-marrow-derived dendritic cells residing in the epidermis [9,10].

In adult patients, LCH is frequently overlooked, as malignancy is commonly the initial suspicion upon finding a lytic lesion. Our encounter with an LCH case confined to the bones of a young adult was deemed a valuable experience. We present a case of LCH in an adult, following the SCARE 2023 criteria [19].

## 2 Case presentation

**Fig. 1:**
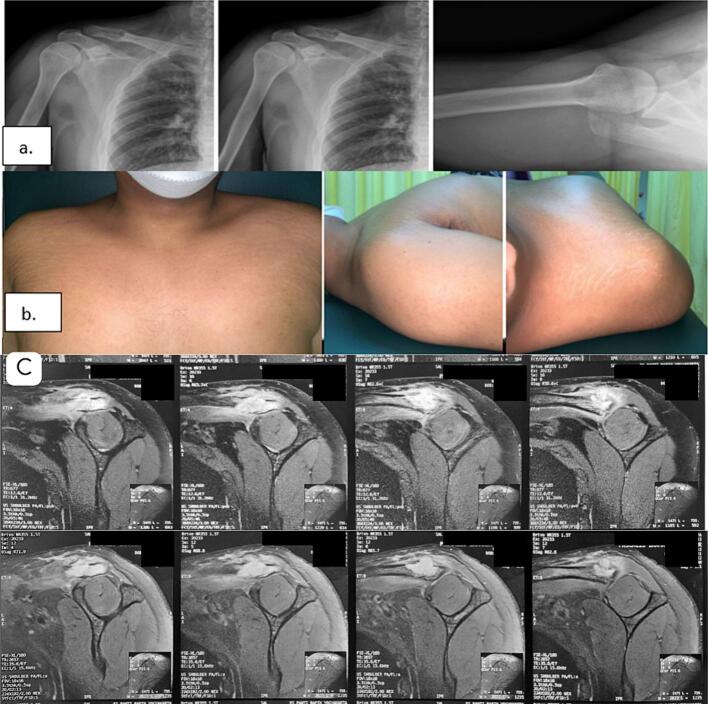
a. Pre-operative radiograph showing lytic lesion on distal clavicle; b. asymmetrical shoulder from; c. MRI with contrast enhancement.

A 21-year-old male was referred to our hospital due to worsening pain in his right shoulder. He denied any history of trauma or excessive load-bearing activities, and there was no family history of cancer. During the physical examination, we observed a deformity in his right shoulder, which appeared asymmetrical compared to the left side, and he experienced pain upon palpation of the distal third of the clavicle. Plain radiographs revealed an osteolytic lesion with cortical thinning in the distal third of the clavicle, while axial and sagittal views of the MRI showed tumor growth on the lateral third of the right clavicle with contrast enhancement (Fig. 1a-c). Before surgery, Multislice-Computed Tomography (MSCT) scans of the abdomen, and thorax, and a complete bone survey were conducted, revealing no additional masses in other regions. Based on the clinical and radiological results, we suspected a primary benign bone lesion with a Bone RADS score of 1, in accordance with the guidelines of the American College of Radiology. We planned for an open biopsy along with curettage and internal fixation of the clavicle which was performed three days later.

**Fig. 2:**
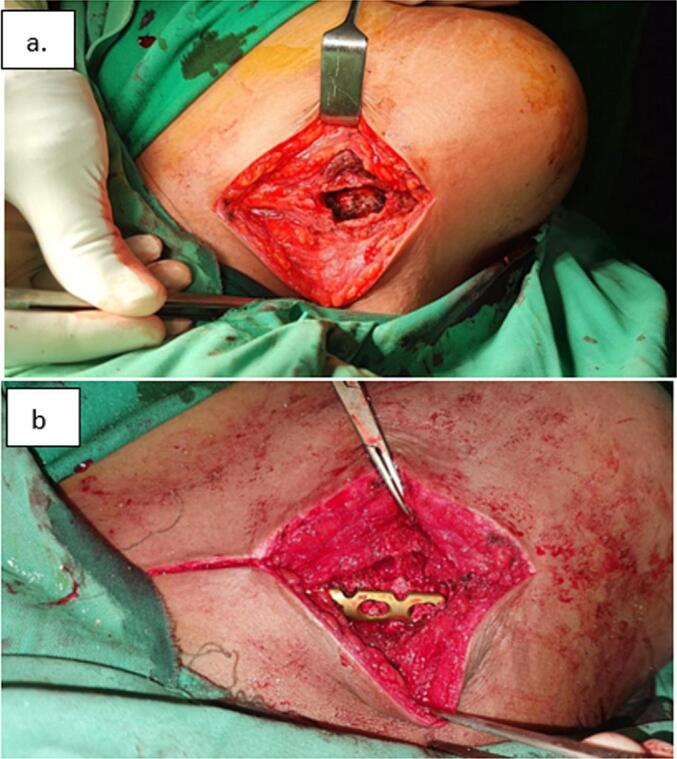
a-b Lesion curettage and fixation and our approach to excised the lesion.

During the surgery, we used the anterior clavicle approach to remove the tumor. We confirmed that there was no fracture. The tumor was sent for diagnosis and the cavity left was filled with bone graft and fixed with a 6-hole plate with a locking mechanism (Fig. 2).

**Fig. 3:**
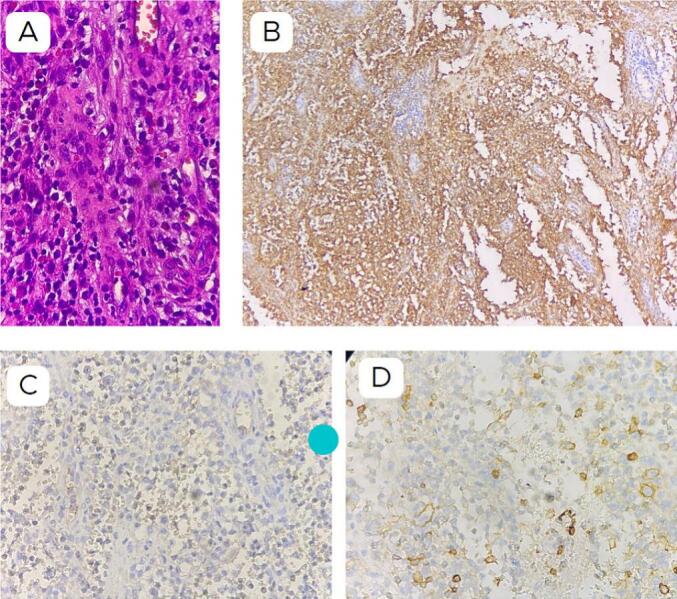
Histopathological result a. Haematoxylin eosin staining, b. IHC with CD1a showed positive results, c. IHC with CD30 showed negative results, d. IHC with CD138 showed a negative result. We conclude the diagnosis was LCH.

Microscopic examination revealed bone tissue with diffuse tumor cells, of moderate size, with eosinophilic cytoplasm. Some cells exhibited oval nuclei, some eccentric, some showing grooves. Among them were numerous eosinophils and lymphocytes, as well as multinucleated osteoclastic giant cells. We proceeded with Immunohistochemistry staining, which yielded positive results for CD1a (+) and negative results for LCA (CD45) (−), CD30 (−), and CD138 (−). Therefore, based on these findings, a diagnosis of Langerhans cell histiocytosis was concluded (Fig. 3).

**Fig. 4:**
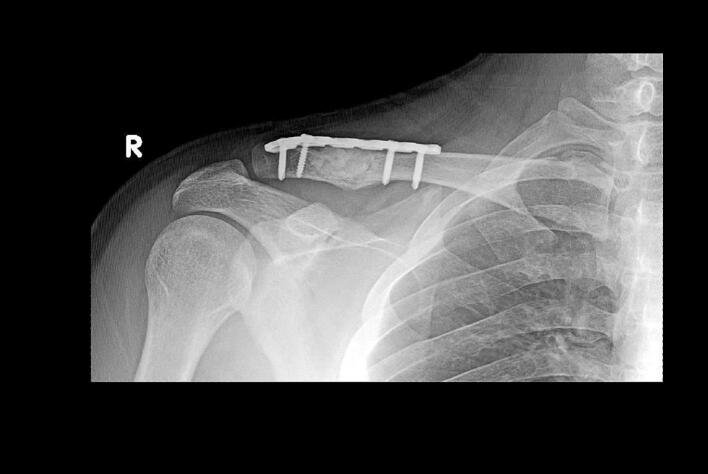
Post-operative radiographs showed good alignment with minimal but increased callous formation. No recurrent mass was seen.

We brought our patient's findings along with our treatment result for clinicopathologic conference with multidisciplinary team, which all agreed on LCH to be the diagnosis. On his nine-month follow-up, no tumor-progressive changes were seen on examination nor complained by the patient (Fig. 4). However, the callous formation has been minimal which concerned us for potentially impaired bone healing. The patient has then received radiotherapy and his latest MRI only showed no residual mass and excellent clavicle alignment.

## 3 Discussion

LCH, a disease spectrum, commonly presents as a lesion confined to the bone, featuring pathological findings of inflammatory cells and abnormal LCs with positive immunohistochemistry staining for CD1a and/or CD207 or Birbeck granules observed under an electron microscope [11,12]. While LCH predominantly affects children, it also occurs in adults aged 40 years and older, with only a few cases reported annually [3]. A systematic review by Reisi et al. compiled case reports of LCH, highlighting the chest wall as the second most common site for unifocal lesions in adult cases, particularly in the sternum and clavicle [13]. Patients typically first seek orthopedic surgeons due to initial complaints of pain and discomfort around the affected structure.

Imaging plays a crucial role in identifying the underlying cause, as patients often present with non-specific findings of pain and inflammatory changes. On radiographs, EG of the extremities typically appears as a punched-out lytic bone lesion without reactive sclerosis, surrounded by a mass of hyper-vascularized soft tissue [11,12]. However, these findings may suggest alternative differentials such as bone cysts, aneurysmal cysts, sarcoma, multiple myeloma, or bone metastasis [9,11,14]. Recognizing our first diagnostic pitfall, we initially suspected the lesion in our patient to be benign based on the well-marginated osteolytic lesion found but did not consider LCH in our differential diagnoses. A retrospective study by Song et al. reported only one of their study subject presented with solitary bone lesion but all were seen with typical osteolytic without reactive sclerosis from plain radiograph. As skeletal LCH lesions may present with either poorly defined or well-demarcated margins, MRI and CT imaging consistently showed endosteal scalloping regardless of lesion numbers [4]. However, considering the overall rarity of LCH cases, the patient's history, age of 21 years and affected area in the clavicle did not seem to fit the demographic spread.

Biopsy remains the gold standard for confirming the disease spectrum of LCH, with clonal expansion of LCs expressing CD1a being mandatory findings. However, the pathophysiology of LCH remains debated, with questions surrounding whether cellular expansion occurs due to neoplastic or immunologic and inflammatory processes [9,12]. Despite evidence of proto-oncogene mutations in LCH samples, explaining the cell cycle disruption leading to neoplastic growth of LCs, the lytic process occurring in bones is assumed to result from the local secretion of factors such as interleukins and prostaglandins [9,11,12,15,16].

Due to the inconclusive pathophysiology of LCH, targeted treatment becomes challenging. After diagnostic confirmation through biopsy, management of a single lesion of skeletal LCH may involve observation or corticosteroid injection for minimally symptomatic patients, while progressive and painful lesions may require more invasive treatment such as surgical curettage with or without bone grafting. Systemic treatments such as chemotherapy and radiotherapy are reserved for recurring, symptomatic, and/or inaccessible sites [9,11,12,17].

We recognise our approach was dissimilar to recommendations which advised for confirmatory biopsy prior to therapy. Instead, with characteristics of benign tumours found from our patient's imaging, we opted for an open biopsy with simultaneous curettage followed by bone grafting and internal fixation. Several studies have pointed out the risks of biopsy, whether it is necessary if the lesion is pathognomonic and the risk of negatively affecting bone healing and reconstitution of granuloma lesion [18]. With pathologic and IHC confirmation for LCH diagnosis and our patient risk stratification with solitary bone lesion, we expect good prognosis and survival over the next 10 years with a recurrence rate as much as 20 % [11,12]. Although longer follow up period is necessary to evaluate progressivity, the patient is very well satisfied with the treatment outcome. The role of clinicopathological conference at our center has helped us to conclude the diagnosis and treatment plan for this patient. We strongly recommend to schedule multidisciplinary team discussion when ensuring possibility of malignancy prior to performing invasive approach. The rarity of managing LCH case has been a valuable experience for our team which hopefully may bring the diagnosis in mind for oncology orthopedic surgeons in their future practices.

Aside from our success in managing this reported case, we recognise our limitation of almost misdiagnose the condition and treating it before the diagnosis was confirmed. As we learn from this experience, we hope orthopedic surgeons will be more careful especially determining treatment approach as LCH can also manifest as a malignancy with involvement of risk organs and worse prognosis.

## 4 Conclusion

Adult presentation of skeletal LCH is rare and often misdiagnosed. Limitations of studies reporting the incidence frequently resulted in negligence in diagnosis. When clinical and supporting examinations do not point to a specific diagnosis, key principle prior to invasively taking biopsy sample is to ensure the risk of lesion being a primary malignant tumor. Multidisciplinary team discussion allows for more comprehensive patient care in confirming diagnosis and treatment plan, which a rare and intricate case like LCH will require.

## Consent for publications

The patient gave written informed consent regarding the use of their data and photographs for publication of the case report.

Written informed consent was obtained from the patient for publication of this case report and accompanying images. A copy of the written consent is available for review by the Editor- in -Chief of this journal on request.

## Ethical approval

This case report doesn't require ethical approval based on the Universitas Gadjah Mada research ethics committee's guidelines. It focuses on a patient's treatment and medical care, not research. Our institution's ethics committee confirmed that this report aligns with routine clinical practice and doesn't involve experimental interventions or additional data collection. We're ready to provide more information if needed, underscoring our commitment to ethical practices.

## Funding

This research received no external funding.

## Author contribution

Conceptualization (Y.A.P·P., R.M. M.I·N) Methodology (Y.A.P.P., R.M., M.I·N) Investigation (Y.A.P.P., M.I·N) Supervision (Y.A.P.P., R.M., E.K.D., E.E., A.W·P) Validation (Y.A.P.P., R.M., E.K.D., E.E., A.W·P) Writing original draft (Y.A.P.P.,M.I·N) Writing review & editing (Y.A.P.P., R.M., E.K.D., E.E., A.W·P).

## Guarantor

Y.A.P.P.

## Research registration number

N/A.

## Conflict of interest statement

All authors declare that there is no conflict of interest regarding the publication of this report.

## Data Availability

Supporting data will be available upon reasonable request.
